# Docking-Based 3D-QSAR Studies for 1,3,4-oxadiazol-2-one Derivatives as FAAH Inhibitors

**DOI:** 10.3390/ijms22116108

**Published:** 2021-06-06

**Authors:** Agata Zięba, Tuomo Laitinen, Jayendra Z. Patel, Antti Poso, Agnieszka A. Kaczor

**Affiliations:** 1 Department of Synthesis and Chemical Technology of Pharmaceutical Substances with Computer Modeling Laboratory, Faculty of Pharmacy, Medical University of Lublin, PL-20059 Lublin, Poland; 2 School of Pharmacy, University of Eastern Finland, FI-70211 Kuopio, Finland; tuomo.laitinen@uef.fi (T.L.); antti.poso@uef.fi (A.P.); 3 Division of Pharmaceutical Chemistry and Technology, University of Helsinki, FI-00790 Helsinki, Finland; jayendra.patel@helsinki.fi; 4 Department of Internal Medicine VIII, University Hospital Tübingen, DE-72076 Tübingen, Germany

**Keywords:** 3D QSAR, CoMFA model, CoMSIA model, FAAH inhibitors

## Abstract

This work aimed to construct 3D-QSAR CoMFA and CoMSIA models for a series of 31 FAAH inhibitors, containing the 1,3,4-oxadiazol-2-one moiety. The obtained models were characterized by good statistical parameters: CoMFA Q^2^ = 0.61, R^2^ = 0.98; CoMSIA Q^2^ = 0.64, R^2^ = 0.93. The CoMFA model field contributions were 54.1% and 45.9% for steric and electrostatic fields, respectively. In the CoMSIA model, electrostatic, steric, hydrogen bond donor, and hydrogen acceptor properties were equal to 34.6%, 23.9%, 23.4%, and 18.0%, respectively. These models were validated by applying the leave-one-out technique, the seven-element test set (CoMFA r^2^_test-set_ = 0.91; CoMSIA r^2^_test-set_ = 0.91), a progressive scrambling test, and external validation criteria developed by Golbraikh and Tropsha (CoMFA r^2^_0_ = 0.98, k = 0.95; CoMSIA r^2^_0_ = 0.98, k = 0.89). As the statistical significance of the obtained model was confirmed, the results of the CoMFA and CoMSIA field calculation were mapped onto the enzyme binding site. It gave us the opportunity to discuss the structure–activity relationship based on the ligand–enzyme interactions. In particular, examination of the electrostatic properties of the established CoMFA model revealed fields that correspond to the regions where electropositive substituents are not desired, e.g., in the neighborhood of the 1,3,4-oxadiazol-2-one moiety. This highlights the importance of heterocycle, a highly electronegative moiety in this area of each ligand. Examination of hydrogen bond donor and acceptor properties contour maps revealed several spots where the implementation of another hydrogen-bond-donating moiety will positively impact molecules’ binding affinity, e.g., in the neighborhood of the 1,3,4-oxadiazol-2-one ring. On the other hand, there is a large isopleth that refers to the favorable H-bond properties close to the terminal phenoxy group of a ligand, which means that, generally speaking, H-bond acceptors are desired in this area.

## 1. Introduction

The fatty acid amide hydrolase enzyme (FAAH) belongs to the serine hydrolase superfamily. It is involved in the degradation of biologically active lipids—endocannabinoids, e.g., anandamide and 2-arachidonoylglycerol—or related-amidated signaling lipids. FAAH activity is considered to play an essential role in the development of multiple pathological conditions [1]. Enzyme inhibitors may exhibit analgesic, anti-inflammatory, anxiolytic, and antidepressant activity. Importantly, blockade of FAAH does not cause undesirable side effects of direct cannabinoid agonists [2]. Due to that fact, its blockade became an emerging strategy in the treatment of several central nervous system (CNS) and peripheral diseases [1,2,3,4]. The development of novel effective FAAH inhibitors became a key focus in drug design [4,5]. Unfortunately, a ligand that recently got into phase 1 of clinical trials resulted in the death of one healthy volunteer and led to some mild-to-severe neurological symptoms in others [6].

Computation resources are widely used in the examination of various properties of medicinal compounds. Quantitative structure–activity relationship methods require building mathematical or computational models to find a significant correlation between the structure and the biological activity of certain groups of substances [7]. These approaches quantitatively correlate the relationships between the chemical structure alterations and respective changes in bioactivity. Their usage enables us to optimize properties of currently used chemicals and predict various parameters that refer to the biological activity of untested and sometimes unavailable compounds [7,8].

The CoMFA (Comparative Molecular Field Analysis) method is considered an advantageous 3D-QSAR approach that has been successfully implemented in many medicinal chemistry studies. This technique stands out from classical QSAR strategies primarily due to its numerous advantages. Significant advantages of this technique include direct applicability in the examination of any structure-dependent biological property. It can be used with almost any set of compounds that can be appropriately aligned and have an activity range spanning over at least three orders of magnitude. Moreover, each CoMFA parameter covers the interaction energy of the whole molecule [7]. In general, it involves statistical methods to correlate both the electrostatic and steric properties of examined molecules with their pharmacological activity. The results of this calculation can be viewed as a 3D-contour map, which presents forces that surround a series of studied, properly aligned molecules. To thoroughly investigate the structure–activity relationship among a set of given compounds, it is also suggested to use the Comparative Molecular Similarity Indices Analysis (CoMSIA). The CoMSIA method, like CoMFA, is based on the structural alignment of compounds but uses atom-centered Gaussian-based fields to describe compounds. In addition, CoMSIA utilizes three additional descriptors (hydrophobic, H-bond donor, H-bond acceptor) [8].

**Figure 1:**
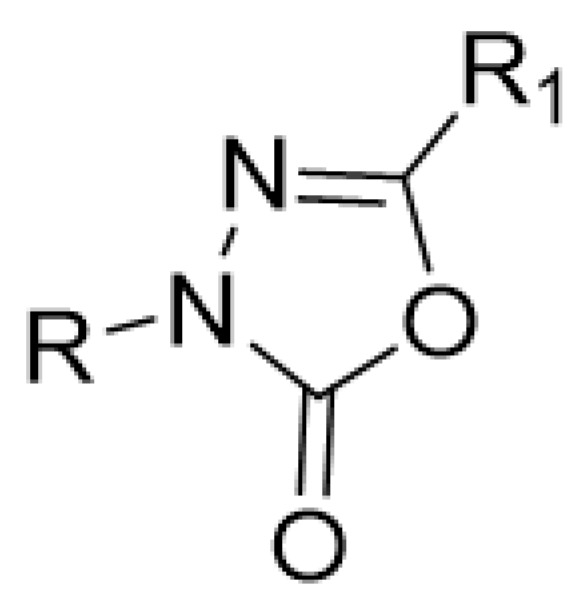
The general structure of the studied compounds.

Unfortunately, there is minimal information about the structure–activity relationship studies performed on a series of different types of fatty acid amide hydrolase inhibitors [9]. Käsnänen et al. synthesized a series of meta-substituted phenolic N-alkyl/aryl carbamates. They later submitted these compounds into the 3D-QSAR analysis, which revealed some interesting structure–activity correlations, e.g., presence of a meta-substituted phenyl ring positively influenced the biological activity of these compounds [10]. Zhao et al. constructed a pharmacophore model for FAAH antagonists based on the set of 21 typical compounds available in the literature. It contained four essential features—two H-bond acceptor units, a hydrophobic part, and one aromatic ring unit. This model was successfully applied to the prediction of the activity of 55 compounds [11]. Later, Han et al. decided to evaluate a series of 26 novel oleoylethanolamide derivatives using the CoMFA method [12]. That provided information about the desirable and unfavorable modifications which can be applied to enhance or decrease the biological activity of these molecules, e.g., a long aliphatic carbon chain enables the molecule to reach a green region of desired steric interactions and increases its potency. The most recent paper published by Lorca et al. described a 3D-QSAR study performed on a series of 90 reported FAAH inhibitors that shared a common structural pattern—pyrimidinyl-piperazine-carboxamide moiety. Structure–activity conclusions obtained from the contour map analysis contributed to the designing of new compounds that showed promising predicted activities [13]. However, we would like to emphasize that our study was designed to investigate the structure–activity relationship among a series of FAAH ligands presented in previously published papers [14,15]. Therefore, the study aimed at investigating the contribution of specific chemical moieties in the development of novel biologically active FAAH inhibitors. Thus, the modeling set comprised only compounds containing the 1,3,4-oxadiazol-2-one moiety, synthesized by Patel et al. [14,15]. Among the diverse scaffolds utilized for the development of FAAH inhibitors, 1,3,4-oxadiazol-2-one has gained recent attention in the development of serine hydrolase inhibitors, including FAAH [9,14,16,17,18,19,20,21,22]. The modeling set comprised novel 1,3,4-oxadiazol-2-ones derivatives (Table 1), previously tested for their inhibitory activity against FAAH [14,15,19,20]. Promising results obtained in these experiments became an incentive to examine the structure–activity relationship among these ligands thoroughly. It is noteworthy that, despite the extensive research that has been carried out on the FAAH itself, no single study exists which adequately examines the structure–activity relationship among FAAH ligands that contain the 1,3,4-oxadiazol-2-one moiety (Figure 1) [14,15]. Due to all these reasons, we decided to take advantage of the availability of the FAAH X-ray structure (PDB ID: 3QK5) [23] and correlate structural and biological properties of the FAAH inhibitors using the 3D-QSAR techniques based on the molecular docking alignment. In our opinion, this work constitutes a novel extension to the previously published papers and will contribute to the development of, more potent compounds.

## 2. Results and Discussion

### 2.1. The Studied Compounds

The studied compounds (**1**–**31**) possessing inhibitory activity against the FAAH enzyme accompanied by their pIC_50_ values (experimental, predicted, and residual) are presented in Table 1. Records in that table were ordered and numbered according to the decreasing pIC_50_ values of the compounds.

The compounds were divided into a training set (24 compounds) and a test set (7 compounds). Moreover, all molecules used in this study showed low residual values, which means the deviation between predicted and experimental values of pIC_50_ for each molecule was lower than one logarithmic unit.

The CoMFA model contains 12 compounds with negative residual values and 12 molecules with positive deviations, whereas the CoMSIA model is divided into groups of 11 and 13, respectively.

### 2.2. Molecular Docking and Alignment

Compounds labeled **1**–**31** were docked with the Glide module from Schrödinger suite v 2018-2 to the binding site of the fatty acid amide hydrolase. The X-ray structure of FAAH in a complex with a small molecule inhibitor (PDB ID: 3QK5) [23] was used for molecular docking. The RMSD value obtained for the superimposition of the re-docked ligand onto the original RCSB PDB’s complex structure was equal to 0.181 Å.

The molecular alignment is one of the most critical aspects that determines the effectiveness of the 3D-QSAR methods. Appropriate poses selection in the docking-based alignment is a key factor affecting the obtained model’s statistics [24,25]. The most important inhibitory contact comprises a hydrogen bond formed between an inhibitor carbonyl group and Ser 241 from the FAAH enzyme. However, we did not perform a constrained docking in order to preserve the complete flexibility of a ligand and an enzyme, which means we did not consider a covalent bond in this position, as reported earlier [14,15]. Among the obtained poses, those identified with high docking scores and interaction with Ser 241 were taken into the next step of this study. Moreover, a consistent alignment of the 1,3,4-oxadiazol-2-one moiety for all ligands was ensured.

### 2.3. CoMFA Statistics and Validation

The CoMFA studies were performed using the QSAR module in the Sybyl-X v 2.1 software in the Windows environment. Among the obtained models, only one met all of the requirements. This CoMFA model was characterized by the optimal number of components of 4 and a high value of R^2^ of 0.98. Moreover, the established model had a cross-validated coefficient of Q^2^ of 0.61 and an F-value of 234.68. The number of components was designated so that the standard error of prediction was minimal, and cross-validated Q^2^ values were maximal. The summary of this procedure, along with the CoMSIA modeling results, are gathered in Table 2.

**Figure 2:**
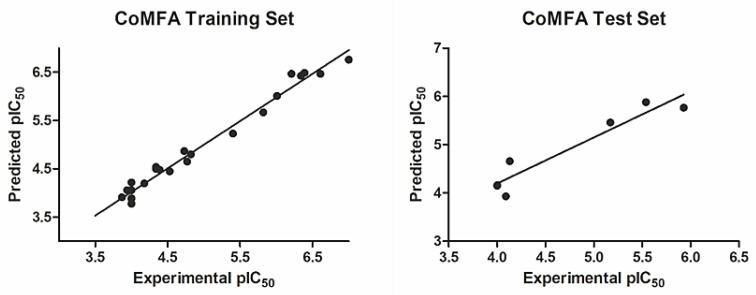
The experimental versus predicted pIC_50_ values for the CoMFA training set and test set.

The experimental, calculated, and residual values of pIC_50_ for the training set and the test set are shown in Table 1; these values do not vary more than the logarithmic unit and thus support the CoMFA model’s statistical validity. Figure 2 shows data correlation between the experimental and the predicted pIC_50_ values for both the training and the test set of compounds.

As an additional method of validation, a scrambling stability test was performed. This test was carried out to find an ideal number of components and check whether the CoMFA model is sensitive to small perturbations applied to the data. In stable models, the value of dQ^2^/dR^2^yy should not exceed 1.2. Moreover, it is considered that an ideal value of this parameter equals 1 [26,27,28]. Obtained results are presented in Table 3.

It is noteworthy that in our model, both parameters (Q^2^ and DQ^2^/dR^2^yy) are within the established ranges, which stand for the high robustness of this CoMFA model.

Furthermore, the CoMFA model was used to predict the pIC_50_ values for the seven-element test set of compounds. All of the parameters obtained in this procedure are also presented in the test-set section of Table 1. Obtained predictions are quite similar to those experimentally determined. A simple regression-based r^2^_test-set_ value of 0.91, which was calculated for the test-set molecules, is another significant proof of the obtained model’s statistical importance.

In the end, our model was examined with the use of external validation criteria proposed by Golbraikh and Tropsha [28,29] (Table 2). It fulfills all of those requirements. It confirms that the proposed CoMFA model is statistically significant and robust.

### 2.4. The CoMFA Contour Map and Its Mapping onto Receptor Structure

Unlike the 2D-QSAR calculations, the CoMFA results can be graphically viewed as a set of colorful plots surrounding the examined compounds. In this technique, there are two types of contributions that can be visualized around the compound. The first one, represented by yellow and green plots, determines regions where the steric interactions occur. In contrast, the second one, which is depicted as red and blue polyhedrons, is related to the electrostatic forces surrounding the examined molecule. Proper examination of these interactions is a valuable source of knowledge, which can provide us with ideas on structure modifications that can be implemented in other drug-design processes.

**Figure 3:**
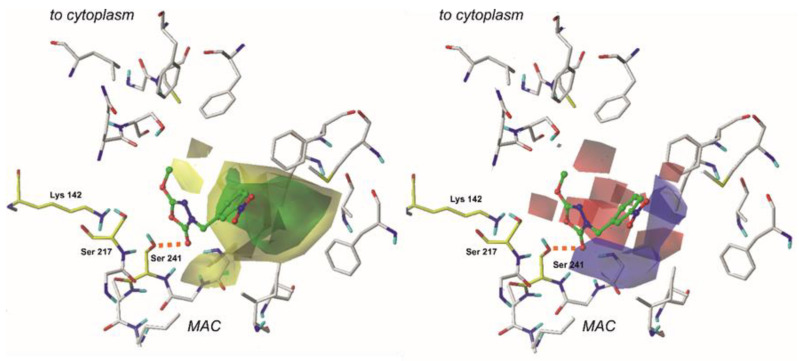
Comparative Molecular Field Analysis (CoMFA) electrostatic (right) and steric (left) fields projected on the 3D structure of the fatty acid amide hydrolase in complex with the least active compound (**31**). Fields are drawn with 70/30 proportions. Ligands are depicted as sticks and balls with green carbon atoms. Protein is shown as sticks with gray carbon atoms. Catalytic residues are presented as sticks and are colored using yellow carbon atoms. A bond formed between a ligand and an enzyme is depicted as an orange dashed line. Non-polar hydrogens are omitted for clarity.

**Figure 4:**
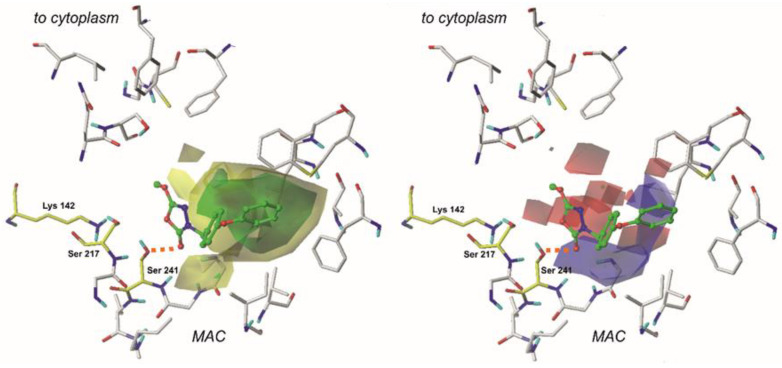
Comparative Molecular Field Analysis (CoMFA) electrostatic (right) and steric (left) fields projected on the 3D structure of the fatty acid amide hydrolase in complex with the most active compound (**1**). Fields are drawn with 70/30 proportions. Ligands are depicted as sticks and balls with green carbon atoms. Protein is shown as with gray carbon atoms. Catalytic residues are presented as sticks and are colored using yellow carbon atoms. A bond formed between a ligand and an enzyme is depicted as an orange dashed line. Non-polar hydrogens are omitted for clarity.

The constructed CoMFA model was characterized by the field fractions representing steric (54.1%) and electrostatic (45.9%) regions. Figure 3 depicts steric and electrostatic contour maps viewed on the least active (no. 31, pIC_50_ = 3.87) compound, and Figure 4 visualizes steric and electrostatic field plots mapped on the most active molecule from the training set (no. 1, pIC_50_ = 7.00) [8,26].

Finally, CoMFA contour maps were generated, and field values were calculated at each point of the three-dimensional grid box. These values contribute a scalar product of the connected QSAR coefficient and the standard-deviation in the associated part of the data plotted as the percentage contributors to QSAR equation [30].

Electrostatic properties are depicted as red and blue regions. Red plots determine regions where moieties with a negative charge are more favorable for enhancing the molecule’s binding affinity. On the other hand, blue fields contribute to the areas where the negative charge is highly unfavorable [7,31].

In the case of each compound from the training set, the examination of electrostatic properties revealed red plots surrounding the 1,3,4-oxadiazol-2-one moiety. This highlights the importance of heterocycle, highly electronegative substituent in this area of each ligand. Moreover, this can explain why compounds **9**, **30**, and **31** that contain aromatic substituents (e.g., benzene ring) with more homogenous charge distribution attached in the red-plot region are generally characterized by lower biological activity. In the case of compound **1,** the blue plots surround the terminal aromatic ring, and it indicates that electropositive constituents are generally more favored in this region. This explains the high biological activity of compounds, e.g., **2** and **10**. Moreover, the presence of the -NO_2_ group in the area encompassed by a blue plot can be considered a possible cause of the low biological activity of compound **31**.

Steric properties are visualized as green and yellow plots. The first one represents regions where the presence of bulky substituents is considered to influence the activity of the compound positively. In contrast, the latter stands for the molecule regions, where extensive moieties are related to decreasing binding [31]. Analysis of steric contour maps, viewed on the compounds from the training set, revealed that high-volume moieties, e.g., aromatic rings, are generally more desired on the opposite side to the 1,3,4-oxadiazol-2-one moiety. Thus, providing these regions with an additional 5- or 6- membered ring might positively influence the binding affinity. It is worth noting that molecules containing small-volume substituents attached to the 3rd position of the mentioned earlier heterocycle do not reach the green region of favorable interactions and are characterized by a lower biological activity.

The presence of several smaller yellow contours surrounding the rest of the compound suggests that the compound’s biological activity decreases as the volume of the moiety attached to the, e.g., 1,3,4-oxadiazol-2-one ring rises. Presumably, the presence of these unfavored interactions can explain the low biological activity of compound **12**. Moreover, providing the ligand with a six-membered ring or another bulky substituent in this area would be highly unfavorable for the activity of this molecule.

### 2.5. CoMSIA Model Statistics and Validation

The same 24-element training set was used to develop a Comparative Molecular Similarity Indices Analysis (CoMSIA) model. All of the obtained parameters are presented in Table 2, along with the CoMFA model statistics. In general, this model was characterized by high values of R^2^ of 0.93 and Q^2^ of 0.64. The optimal number of components was equal to 3. This model was also characterized by a low value of the standard error of prediction equal to 0.28. The non-cross validated PLS (partial least square) analysis resulted in an F-value of 92.82.

**Figure 5:**
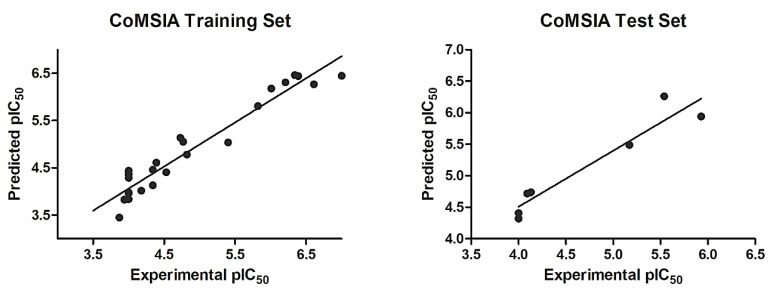
The experimental versus predicted pIC_50_ values for the CoMSIA training set and test set.

At first, this model was evaluated with the use of the PLS leave-one-out (LOO) procedure. The Q^2^ value of 0.64 obtained by this method stands for the high stability of the model. As the next step in validation, our model was used to predict the pIC_50_ values for compounds in the seven-element test set, and it revealed a high correlation of the data (_r_^2^_test-set_ = 0.91). Figure 5 depicts data correlation values for both the training set and the test set.

We used a scrambling stability test to generate additional validation parameters and evaluate the statistical significance of the CoMSIA model. The results obtained from this procedure are presented in Table 4. It is noteworthy that for the optimum number of components of 3, the dQ^2^/dR^2^yy value does not exceed 1.2 [26].

Finally, we introduced our model to the external validation criteria proposed by Golbraikh and Tropsha [28,29]. All of the obtained parameters stand within recommended values (Table 2). Thus, all presented data stand for good stability and high statistical significance of the CoMSIA model.

### 2.6. The CoMSIA Model Statistics and Validation

**Figure 6:**
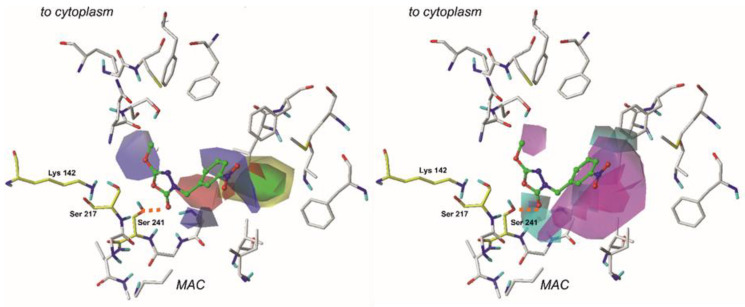
Comparative Molecular Similarity Indices Analysis (CoMSIA) steric, electrostatic (left), H-bond acceptor and H-bond donor (right) fields projected on the 3D-structure of fatty acid amide hydrolase in complex with the least active compound (**31**). Fields are drawn with 60/40, 70/30, 60/40, 60/40 proportions, respectively. Ligands are shown as sticks and balls with green carbon atoms. Protein is shown as wires with gray carbon atoms. Catalytic residues are presented as sticks and colored using yellow carbon atoms. A bond formed between a ligand and an enzyme is depicted as an orange dashed line. Non-polar hydrogens are omitted for clarity.

**Figure 7:**
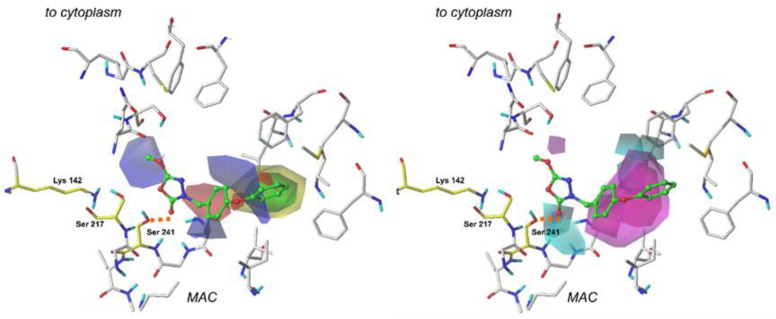
Comparative Molecular Similarity Indices Analysis (CoMSIA) steric, electrostatic (left), H-bond acceptor and H-bond donor (right) fields projected on the 3D-structure of fatty acid amide hydrolase in complex with the most active compound (**1**). Fields are drawn with 60/40, 70/30, 60/40, 60/40 proportions, respectively. Ligands are shown as sticks and balls with green carbon atoms. Protein is shown as wires with gray carbon atoms. Catalytic residues are presented as sticks and colored using yellow carbon atoms. A bond formed between a ligand and an enzyme is depicted as an orange dashed line. Non-polar hydrogens are omitted for clarity.

The CoMSIA 3D-Contour maps were generated in order to visualize the results obtained via these specific calculations. The contour maps of the CoMSIA for the least active and the most active of compounds are depicted in Figure 6 and Figure 7. In the CoMSIA model, the fractions representing electrostatic, steric, hydrogen-bond donor, and hydrogen acceptor fields were 34.6%, 23.9%, 23.4%, and 18.0%, respectively.

Similarly, as in the CoMFA technique, steric and electrostatic contour plots are returned as a result of the CoMSIA modeling. In the case of our CoMSIA model, steric and electrostatic contours obtained in the calculation are in good agreement with the results generated by the previously performed CoMFA study. It is noteworthy that steric and electrostatic interactions’ contribution was relatively smaller than in the previously performed examination [8].

In the CoMSIA study, two additional contour plots are generated—hydrogen bond donor and hydrogen bond acceptor. Their graphical interpretation is depicted in Figure 6 and Figure 7. These fields were generated with 60/40 contribution levels.

Hydrogen bond donor fields are represented by cyan and purple plots. In this context, cyan fields constitute favorable interactions, which means that hydrogen-bond-donating groups in these regions positively affect the molecule’s binding affinity. Purple marks indicate plots where H-bond donors are generally not required, and their presence results in the compound’s biological activity decrease. A large plot showing favorable H-bond donor properties is located in the neighborhood of a terminal aromatic ring in our reference compounds. Hence, providing these structures with another amine or hydroxyl group might benefit the ligand’s binding affinity. It appears that another contour of this type is present close to the 1,3,4-oxadiazol-2-one ring and oxygen atom from the enzyme’s Ser 241 residue. This suggests that the examined area is essential for a binding of a ligand. Moreover, it is believed that introducing another hydrogen-bond acceptor group might positively influence binding affinity and the compound’s biological activity. Interestingly, the same oxygen atom from the carbonyl group (1,2,4-oxadiazol-2-one moiety) of a ligand is involved in other H-bond contacts with Ile 238 and Gly 239. These residues, along with Gly 240, form an oxyanion hole—a characteristic structure responsible for stabilization of intermediates derived in the enzyme’s activation process. Introducing another hydrogen bond donor in this particular place may positively impact the compound’s biological activity.

When studying H-bond acceptor properties, magenta isopleths represent regions where the presence of hydrogen-bond-accepting groups will increase the biological activity of a compound. In contrast, such groups are not desired in fields encompassed by red plots [8]. Several plots need to be considered in this context. In the case of compound **1**, a bulky isopleth indicating H-bond acceptor properties is located in a terminal’s phenoxy group neighborhood, which means that, generally speaking, H-bond acceptors are desired in this area. Thus, providing the structure with more electronegative atoms that can act as H-bond acceptors will positively influence the ligand’s binding affinity. Presumably, in the case of compound **31**, providing the structure with additional groups acting as H-bond acceptors in the fields encompassed by magenta (e.g., instead of terminal -NO_2_ group) would positively influence the molecule’s biological activity.

## 3. Materials and Methods

### 3.1. Selection and Preparation of Compounds

A series of 1,3,4-oxadiazol-2-one derivatives characterized by the IC_50_ values were taken from our previously published papers (Table 1) [14,15]. In compounds with the IC_50_ values, which referred to the racemic mixture, the more active enantiomer (*S*) was selected for further examination.

Moreover, in several compounds, e.g., **17** and **18**, the IC_50_ value was not determined experimentally. However, it was calculated from the IC_50-single_, according to the method previously published by Yamamoto et al. (2004) [32].

Furthermore, for some compounds, e.g., **28** and **29**, with the IC_50_ above 100,000 nM, the measurement method could not detect the parameter’s precise value. Thus, the pIC_50_ value was assumed as 4 [33].

### 3.2. Molecular Docking and Alignment

The set of examined compounds was docked with the Glide module (Glide, Schrödinger software Release 2018-2, Schrödinger, New York, NY, USA) to the X-ray structure of the humanized rat FAAH enzyme in a complex with a small molecule inhibitor (PDB ID: 3QK5) [23]. Although there are many X-ray structures of fatty acid amide hydrolase available in PDB, we decided to choose this particular one due to its high X-ray crystallography resolution of 2.2 Å.

The biomolecule structure was preprocessed using the Protein Preparation Wizard (Protein Preparation Wizard; Epik, Schrödinger, New York, NY, USA) to optimize the hydrogen bonding network and remove any possible crystallographic artifacts as reported previously [14].

The grid box was centered, applying the X-ray ligand as the template. Molecular docking was carried out using the Standard Precision (SP) method, and 50 poses were generated for each ligand. Poses, where inhibitor interacted with the FAAH enzyme via a hydrogen bond with Ser 241, were the only ones taken for further analysis [5].

Moreover, in order to examine the correctness of the utilized docking procedure, the RMSD value was calculated in Yasara (v20.1.2.24, CMBI, Radboud University Nijmegen, The Netherlands). This protocol aimed to investigate whether a small molecule chemical binds exactly to the active site of the fatty acid amide hydrolase. Additional information about this procedure and a graphical interpretation of the results are gathered in Supplementary Figure S1.

### 3.3. CoMFA Studies

A modeling set of 31 compounds was submitted for the CoMFA field calculation. In a final model, the training set contained 24 molecules, whereas the test set comprised seven compounds. Test set molecules were chosen in a manner that allowed to cover a reasonable distribution of the biological data. Examined compounds were aligned in a way that allowed to overlaid essential (for the ligand binding) regions of each structure. The Standard Tripos force field was applied with Gasteiger–Hückel point charges and the default sp^3^ carbon probe with point charge +1.0 [34].

The CoMFA analysis was carried out using the QSAR module available in Sybyl-X (v2.1., Tripos Inc., St. Louis, MO, USA) The final model was designed for the optimal number of components, so the cross-validated R^2^ and Q^2^ were at their maximum, while the standard error of prediction was at its lowest [31,35].

As the first step in validation, the PLS analysis was carried out to correlate CoMFA fields to biological activity values (pIC_50_). Next, the leave-one-out (LOO) method was used to cross-validate the model. In this method, one of the compounds is removed from a dataset. Then, the activity of the missing compound is being predicted using the model developed from the remaining molecules. Moreover, scaling was set to the Standard CoMFA, and the column filtering option was set to 1.0 kcal/mol—to omit those columns where the energy variance was lower than 1.0 kcal/mol [36]. In the next step, the statistical significance of the developed model was examined using a seven-element test set of compounds.

A thorough validation procedure also included the usage of a progressive scrambling stability test. This test was performed to find an ideal number of components and check whether the CoMFA model is sensitive to the small perturbations, which were applied to the data [26,35]. As an additional method of validation, the constructed 3D-QSAR model was examined with the use of external criteria, which were previously implemented by Golbraikh and Tropsha [28,29]. Due to these requirements, a model can be considered predictive and statistically significant if the following conditions are satisfied:

Q^2^ > 0.5; r^2^_test-set_ > 0.6; (r^2^ − r^2^_0_)/r^2^ < 0.1; 0.85 ≤ k ≤ 1.15

The r^2^ _test-set_ and r^2^_0_ are squared correlation coefficients between experimental and predicted biological activity values for the test set molecules with and without Y-intercept set to 0, respectively. *‘k’* is a parameter that refers to the slope of the regression line [37].

The CoMFA contour maps were mapped onto the binding site of the FAAH enzyme, and the structure–activity relationship was discussed in the context of protein–ligand interactions [7,31].

### 3.4. CoMSIA Studies

The same 24 element training set was also used to construct the CoMSIA model. The model was built in order to examine the structure–activity relationship among FAAH ligands thoroughly. Both the CoMFA and CoMSIA techniques are based on the assumption that there are correlations between changes in a molecule’s binding affinity and properties expressed as molecular fields [11,21].

In this study, the CoMSIA model was created using Sybyl-X (v2.1., Tripos Inc., St. Louis, MO, USA), and the attenuation factor was set to the default value of 0.3 [38]. The grid constructed for the CoMFA analysis was also used for this calculation. The sp^3^-hybridized carbon atom, with +1.0 probe charge, hydrogen bond donor, and acceptor properties, were placed at each grid point to measure four physicochemical properties (electrostatic, steric, hydrogen-bond donor, and H-bond acceptor). Similarly, positions outside and inside molecular surfaces were calculated at all grid points, while the Gaussian function was applied to determine the distance between molecule and probe atoms [39]. The hydrophobic field was omitted.

The evaluation of this model was performed using the same methods that were earlier applied to determine the CoMFA model’s statistical significance. The CoMSIA model was obtained with the use of the partial least square (PLS) technique. In this calculation, the CoMSIA fields were used as independent variables, whereas values of the pIC_50_ of each compound were treated as dependent variables. Next, the leave-one-out (LOO) approach was used to select the best out of the established models and generate the cross-validated value of R^2^ (Q^2^) and the optimum number of components. Moreover, the PLS analysis was performed for the optimum number of components to determine correlation coefficient R^2^, standard error of prediction, and F-value. Due to this procedure, it was possible to obtain the model characterized by the optimal number of components, corresponding cross-validated Q^2^ value, and the lowest cross-validated standard error of estimate [11].

To examine the computed model’s statistical significance, we used it to predict pIC_50_ values for the seven-element test set. Next, a scrambling stability test was performed as an additional method of evaluation. The CoMSIA model was also evaluated using parameters determined by Golbraikh and Tropsha [28,29]. Finally, the CoMSIA results were graphically interpreted as colorful contribution maps.

## 4. Conclusions

We used 3D-QSAR techniques to examine the structure–activity relationship of a series of 1,3,4-oxadiazol-2-one compounds. Both constructed 3D-QSAR models were derived from a modeling set containing 31 compounds. Moreover, they were evaluated using the same statistical methods, including the leave-one-out technique, prediction of pIC_50_ values for an external group of compounds, scrambling stability test, and additional external validation criteria presented by Golbraikh and Tropsha. Obtained results stand for a high statistical significance of these models. The CoMFA and the CoMSIA contour maps provided enough information to understand the structure–activity relationship and identify structural features influencing the inhibitory activity. In particular, these compounds interacted with other residues, such as Gly 239 and Ile 238, that seem to be essential for inhibitor binding. The examination of the electrostatic properties of established models revealed plots referring to the desired electronegative groups, surrounding the 1,3,4-oxadiazol-2-one moiety. This highlights the importance of heterocycle, a highly electronegative moiety in this area of each ligand.

Moreover, analysis of steric contour maps, displayed on the compounds from the training set, revealed that high-volume moieties, e.g., aromatic rings, are generally more desired on the opposite side to the 1,3,4-oxadiazol-2-one moiety. Thus, providing these regions with an additional 5- or 6-membered ring might positively influence the binding affinity. Examination of hydrogen bond donor and acceptor properties contour maps revealed several spots where the implementation of another hydrogen-bond-donating moiety will positively impact molecules’ binding affinity, e.g., in the neighborhood of the 1,3,4-oxadiazol-2-one ring. On the other hand, providing the structure with an additional moiety that would act as a H-bond donor in the neighborhood of the terminal phenoxy group may positively influence the molecule’s properties.

The data presented in this manuscript fill the gap in the research on the FAAH inhibitors. In our opinion, this study contributes to a better understanding of a complex structure–activity relationship present among these ligands. Therefore, this information can be used by medicinal chemists to design novel, more potent and selective FAAH inhibitors to treat patients suffering from a number of diseases, e.g., Parkinson’s disease or schizophrenia.

## Figures and Tables

**Table 1:** Modeling set (the training set and the test set) used in the 3D-QSAR study.

No.	Chemical Structure	Experiment pIC_50_	CoMFA	CoMSIA	Residual CoMFA	Residual CoMSIA
Training Set						
1	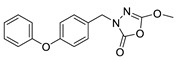	7	6.76	6.45	0.24	0.55
2	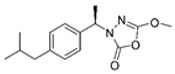	6.61	6.47	6.27	0.14	0.34
3	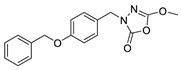	6.39	6.49	6.44	−0.10	−0.05
4	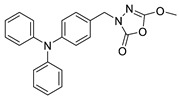	6.34	6.43	6.46	−0.09	−0.12
5	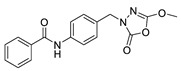	6.21	6.47	6.31	−0.26	−0.10
6	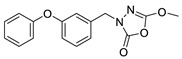	6.01	6.01	6.18	0.00	−0.17
8	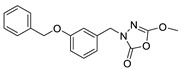	5.82	5.67	5.81	0.15	0.01
10	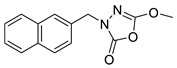	5.40	5.23	5.04	0.17	0.36
12	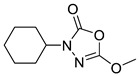	4.82	4.80	4.78	0.02	0.04
13	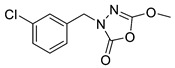	4.77	4.65	5.05	0.12	−0.28
14	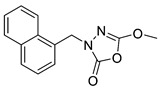	4.73	4.87	5.14	−0.14	−0.41
15	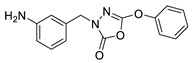	4.53	4.45	4.41	0.08	0.12
16	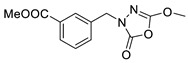	4.39	4.48	4.61	−0.09	−0.22
17	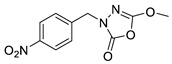	4.34	4.50	4.13	−0.16	0.21
18	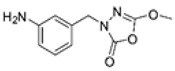	4.34	4.54	4.46	−0.20	−0.12
19	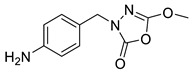	4.18	4.20	4.02	−0.02	0.16
22	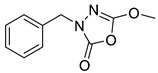	4.00	3.88	3.96	0.12	0.04
23	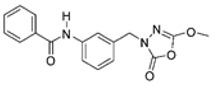	4.00	3.89	4.37	0.11	−0.37
25	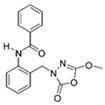	4.00	4.06	3.98	−0.06	0.02
26	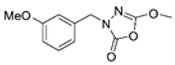	4.00	4.22	4.29	−0.22	−0.29
28	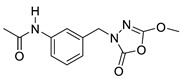	4.00	3.89	3.84	0.11	0.16
29	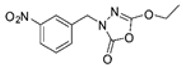	4.00	3.78	4.44	0.22	−0.44
30	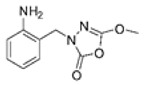	3.94	4.06	3.83	−0.12	0.11
31	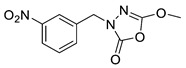	3.87	3.91	3.45	−0.04	0.42
Test Set						
7	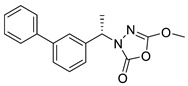	5.93	5.77	5.94	0.16	−0.01
9	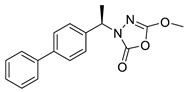	5.54	5.88	6.26	−0.34	−0.72
11	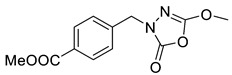	5.17	5.46	5.49	−0.29	−0.32
20	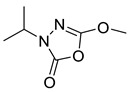	4.13	4.66	4.74	−0.53	−0.61
21	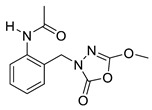	4.09	3.93	4.72	0.16	−0.63
24	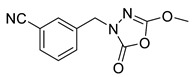	4.00	4.15	4.32	−0.15	−0.32
27	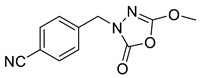	4.00	4.16	4.41	−0.16	−0.41

**Table 2:** Statistical analysis results for the best CoMFA and CoMSIA models.

Parameter	R^2^	Q^2^	SEE	F-Value	Components	r^2^_test-set_	r^2^_0_	k	(r^2^ − r^2^_0_)/r^2^
CoMFA	0.98	0.61	0.16	234.68	4	0.91	0.98	0.95	0.08
CoMSIA	0.93	0.64	0.28	92.82	3	0.91	0.98	0.89	0.08

**Table 3:** Progressive scrambling test results obtained for the comparative molecular fields analysis (CoMFA) model.

Number of Components	5	4	3	2	1
Q^2^	0.51	**0.51**	0.47	0.45	0.34
cSDEP	0.80	**0.78**	0.70	0.79	0.84
DQ^2^/dR^2^yy	1.12	**0.99**	1.04	0.77	0.78

**Table 4:** Progressive scrambling test results obtained for the comparative molecular similarity indices analysis (CoMSIA) model.

Number of Components	5	4	3	2	1
Q^2^	0.47	0.50	**0.51**	0.46	0.32
cSDEP	0.83	0.79	**0.76**	0.79	0.86
DQ^2^/dR^2^yy	1.54	1.28	**1.18**	1.01	0.78

## Data Availability

Supporting information for this article is available upon request from corresponding authors.

## Supplementary Materials

The following are available online at https://www.mdpi.com/article/10.3390/ijms22116108/s1, Figure S1: Three-dimensional graphical interpretation of the docking validation procedure.

## Abbreviations

CoMFA	Comparative Molecular Field Analysis
CoMSIA	Comparative Molecular Similarity Indices Analysis
FAAH	Fatty Acid Amide Hydrolase
MAC	Membrane Access Channel
PLS.	Partial Least Square
QSAR	Quantitative Structure–Activity Relationship
SEE	Standard error of estimate
SP	Standard Precision
