# Supplementary material for: Docking-Based 3D-QSAR Studies for 1,3,4-oxadiazol-2-one Derivatives as FAAH Inhibitors
Source: Int J Mol Sci. 2021 Jun 6;22(11):6108. doi: 10.3390/ijms22116108 (PMC8201265; doi:10.3390/ijms22116108)
Supplement: Supplementary file 1 [file ijms-22-06108-s001.zip › ijms-1206256-supplementary.pdf]

Supplementary Materials

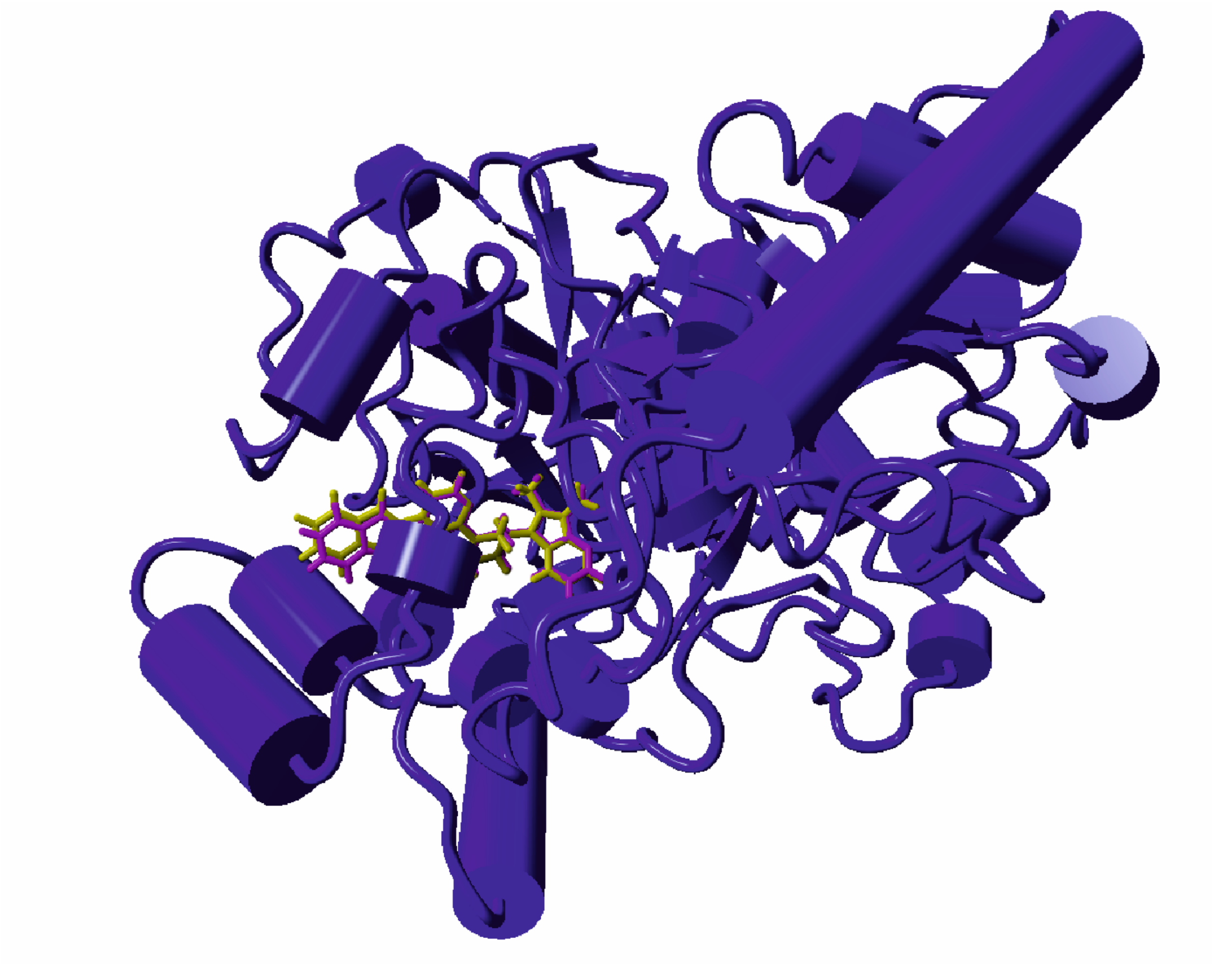

Figure S1. Three-dimensional graphical interpretation of the docking validation procedure. Protein shown in blue in cartoon representation. Crystallized ligand shown in magenta in stick representation. Re-docked pose of the ligand shown in yellow in stick representation.

In order to examine the correctness of the utilized docking procedure, we decided to perform it's validation. The QK5 ligand ((3-{(3R)-1-[4-(1-benzothiophen-2-yl)pyrimidin-2-yl]piperidin-3-yl}-2-methyl-1H-pyrrolo[2,3-b]pyridin-1-yl)acetonitrile was removed from the original fatty acid amide hydrolase's Protein Data Bank entry (PDB ID: 3QK5). The compound was re-docked onto the previously created grid file based on the fatty-acid- amide hydrolases structure. This validation procedure was carried out with the use of the standard precision method available in the Glide module from Schrodinger's suite. The best docking pose was superimposed on the original RCSB PDB entry with Yasara and the RMSD score was calculated (0.181 Å).
